# A population-based survey for dietary patterns and prediabetes among 7555 Chinese adults in urban and rural areas in Jiangsu Province

**DOI:** 10.1038/s41598-020-67028-z

**Published:** 2020-06-26

**Authors:** Ye Cao, Chong Chen, Lan Cui, Aohan Han, Qingyun Tu, Peian Lou, Ganling Ding, Yu Qin, Quanyong Xiang

**Affiliations:** 1 School of Public Health, Southeast University, 87 Dingjiaqiao Road, Nanjing, Jiangsu Province 210009 P.R. China; 2 Department of Chronic Non-communicable Disease Control, Jiangsu Provincial Center for Disease Control and Prevention, 172 Jiangsu Road, Nanjing, Jiangsu Province 210009 China; 3 Department of Chronic Non-communicable Disease Control, Xuzhou Center for Disease Control and Prevention, 142 Erhuanxi Road, Xuzhou, Jiangsu Province 221006 P.R. China

**Keywords:** Lifestyle modification, Epidemiology, Pre-diabetes, Nutrition

## Abstract

Background: Prediabetes is an important public health problem concern globally, to which dietary patterns have shown varied effects. This study aims to analyze the relationship between dietary patterns and prediabetes in Chinese adults. Methods: A total of 7555 adults from Jiangsu province, China, were recruited using a stratified multistage cluster sampling method. Information on diet intake, demographic, blood glucose and other indices were collected by structured questionnaires. Four dietary patterns of Meat diet, Healthy diet, Traditional diet and Fried food with staple diet were identified using Principle Component Analysis and followingly divided into T1 - T4 groups according to their quartiles of factor scores. Multivariate logistic regression analysis was used to investigate the association between dietary patterns and prediabetes. Results: Healthy diet was found to be associated with the lowest prevalence of prediabetes (*P *< 0.05). Multivariate logistic regression analysis after adjusting the confounding factors demonstrated that the lowest odds ratio with prediabetes was associated with the third quartile (T3 group) of Healthy diet (*Odds Ratio* = 0.745, 95*% Confidence Interval*: 0.645–0.860, *P* < 0.01), compared with the lower quartile (T1 group). The Meat diet was a potential risk factor for the isolated IFG (*Odds Ratio* = 1.227, 95%*Confidence Interval*: 1.070–1.406, P-value<0.01) while Fried food with staple diet was positively linked to the presence of IFG combined with IGT (*Odds Ratio* = 1.735, 95% *Confidence Interval:* 1.184–2.543, *P-value* < 0.01). Conclusions: Dietary patterns rich in meat but low in fresh fruit, fresh vegetable, milk, and fish are positively associated with higher risk of prediabetes, particularly the IFG. Higher Healthy diet consumption was associated with significantly lower risk of prediabetes.

## Introduction

Recent global statistics showed that there were 424.9 million people (20–79 years) living with diabetes and 352.1 million people with impaired glucose tolerance (IGT) while the numbers are expected to be 628.6 million and 531.6 million respectively in 2045^1^. Diabetes is prevalent in China. In 2010, the age-standardized prevalence of diabetes and prediabetes was 9.7% and 15.5% among Chinese people aged 20 years of age or older, equivalent to 92.4 million of adults with diabetes and 148.2 million with prediabetes^2^. Prediabetes refers to the level of glycemic parameters higher than the normal but lower than the diagnostic threshold of diabetes. People with prediabetes is known to be at a higher risk to develop diabetes with an annual conversion rate of 5–10%, and approximately 70% of people with prediabetes may develop diabetes in later life according to the statistics of the American Diabetes Association (ADA) expert panel^3,4^. Prediabetes is a reversible morbidity status and about 5–10% patients may revert back to the normal status if risk factors could be well modified^3^.

Evidence showed that about 90% of new cases of diabetes mellitus were attributable to unhealthy lifestyle factors, including tobacco smoking, physical inactivity, alcohol drinking, and excessive intake of high energy foods^5^. ADA has recommendations on the dietary strategies for the individuals at high risks of type 2 diabetes to reduce intakes of foods with high energy and fat but increase the intakes of foods containing whole grains and dietary fiber, while limit the intakes of sugar-sweetened beverages^6,7^. A meta-analysis result of 10 studies indicated that healthy diets (e.g., less fat and high fiber intake) were inversely associated with the risk of type 2 diabetes, yielding a 32% reduction of type 2 diabetes compared with those with unhealthy dietary habits^8^. A large epidemiological study in Chinese adults indicated that higher fresh fruit consumption was associated with significantly lower risk of diabetes^9^. Tonstad, *et al*. reported that after adjustment related factors, the vegans (OR = 0.51, 95% CI:0.40–0.66), lacto-ovo vegetarians (OR = 0.54, 95% CI: 0.49–0.60), pescovegetarians (OR = 0.70, 95% CI:0.61–0.80), and semi-vegetarians (OR = 0.76, 95% CI: 0.65–0.90) had a lower risk of type 2 diabetes than nonvegetarians^10^. Vegetables and vegetarian diets were associated with a substantial and independent reduction in diabetes incidence^11–13^.

Diabetes is a chronic disease, and those affected are often exposed to long-term polypharmacy; therefore, the reduction in diabetes risk was achieved by reversing prediabetes, regardless of the method used; above all, lifestyle changes early on in the pre-disease state should be emphasized^3^. The impact of diet, especially single type of food, on diabetes has been widely investigated in epidemiological studies, but the association between dietary pattern and prediabetes under study, particularly for the Chinese population. Until now, only one pertinent epidemiological study in China revealed that increased consumption of vegetables- fruits dietary pattern or decreased intake of animal offal-dessert dietary pattern might lower down the level of impaired fasting glucose (IFG) in 1495 Chinese men aged 20–75 years;^14^ whether such an association exists in Chinese women, it is unknown. Therefore, this study aimed at investigating the association of impaired glucose tolerance (IGT) and IFG with various dietary patterns determined by principle component analysis (PCA) among 7555 Chinese men and women in Jiangsu, China.

## Methods

This study consisted of a representative sample of the general population from Jiangsu province, China using a 3-stage probability sampling method, covering major geographic areas of Jiangsu Province during 08/2013–09/2015. The 3-stage probability sampling was based on geographic regions (North, Midland, and South area of Jiangsu Province), urban/rural location, and socioeconomic status. Finally, 8 districts and 6 counties were selected in this study. We further randomly selected 4 subdistricts in urban areas or townships in rural areas from each district or county, and then chose 3 neighborhood communities or administrative villages from each subdistrict or township; and then randomly chose 50 households from each selected neighborhood community or administrative village. Finally, we randomly selected one eligible person who was aged ≥18 years and had been living in their current residence for at least 6 months from each selected household using a Kish selection table^15^. A total of 8400 people participated in this study but 845 of them were excluded as ineligible, including 324 self-reported diabetes, 461 newly diagnosed diabetes and 60 people who have a self-report diabetes and refused to collected the serum samples due to either lack of interest (4 persons) or invalid information of laboratory (8 persons) or having comorbidities (48 persons) (e.g., kidney disease, and/or pancreatic disease; cancer or serious cardio-cerebrovascular disease). Finally, a total of 7555 eligible subjects were included in the data analysis.

Each participant was invited to complete a standard questionnaire including information on demographic characteristics, medical history, and lifestyle factors administered by trained interviewers. Dietary information was collected used the food frequency questionnaire (FFQ) in Chinese population, which included food frequency, amount of intake and types of food according to standard food model to assist the participants to recall food consumption^16–18^. The FFQ included 17 items of food, such as staple foods (rice, flour and other cereals), meat, aquatic products, eggs, soy products, vegetables, fruit, milk and dairy products, and so on. Both food consumption and nutrient intake in the FFQ are highly reproducible with good reliability, and the overall correlation coefficient ranges from 0.23 to 0.90^16,19^, which is better than other studies^17^. Body weight and height were measured in light cloth and without shoes according to a standard protocol^20^; BMI (Body mass index) was calculated as weight in kilograms divided by height in squared meters (kg/m^2^).

Blood pressure was measured at left arm 3 times consecutively according to the standard protocol, with a 1–2 minute interval between the measurements with the participants in a seated position at least 5 minutes rest using an automated device (OMRON Model HEM-7071, Omron Co). Blood samples were collected from all eligible participants after an overnight fasting for at least 10 hours. Participants without a self-reported history of diabetes were given a standard 75 g glucose solution, and the plasma glucose was measured at 0 and 2 hours after administration of the oral glucose tolerance test (OGTT). Plasma glucose was measured in an accredited lab using glucose oxidase or hexokinase methods within 12 hours. All study laboratories successfully completed a standardization and certification program. Serum samples were aliquoted and frozen at −80 °C within 2 hours of collection and transported in dry ice to the central laboratory in Jiangsu Province Center for Disease Control and Prevention, which was certificated by The National Laboratory Certification of China. Serum Total cholesterol (TC), low-density lipoprotein cholesterol (LDL-C), high-density lipoprotein cholesterol (HDL-C), and triglycerides (TG) were measured using an auto-analyser (Abbott Laboratories).

A stringent quality assurance and quality control program was implemented to ensure the validity and reliability of study data. All investigators and research staff underwent a week-long training session on the use of standardized protocols and instruments for data collection. Only certified staffs were allowed to collect data. All laboratory equipment was calibrated and the blinded duplicate samples were used. All data were double entered in a database of Epidata (version 3.5) and check for accuracy. This study protocol was approved by the ethical review committee of the Jiangsu Province Center for Disease Control and Prevention (the committee’s reference number: SL2015-B004-01). Written informed consent was obtained from all study participants. The procedures were in accordance with the standards of the ethics committee of Jiangsu Provincial Center for Disease Control and Prevention and with the Declaration of Helsinki (1975, revised 2013).

### Diagnostic criteria for diabetes/prediabetes and other clinical variables

According to the guidelines for the prevention and treatment of type 2 diabetes in China (2013), the diagnosis criteria of diabetes and prediabetes are defined as the following: (1)diabetes: fasting plasma glucose(FPG) ≥ 7.0 and/or OGTT ≥ 11.1 mmol/L; (2) IFG: 5.6≤FPG ≤ 6.9 mmol/L and OGTT <7.8 mmol/L; (3) IGT: 7.8≤OGTT ≤ 11.0 mmol/L and FPG <5.6 mmol/L; (4)IFG and IGT: 5.6≤FPG ≤ 6.9 mmol/L and 7.8 ≤ OGTT ≤ 11.0 mmol/L^21^. BMI (kg/m^2^) of participants were classified into four categories according to the national criteria of body weight for Chinese adults (WS/T 428-2013): light (BMI ≤ 18.49 kg/m^2^), normal (18.50 ≤  BMI ≤ 23.99 kg/m^2^), overweight (24.00≤BMI ≤ 27.99 kg/m^2^), and obesity (BMI ≥ 28.00 kg/m^2^)^22^. Hypertension was defined as systolic blood pressure (SBP) ≥ 140 mmHg and/or diastolic blood pressure(DBP) ≥ 90 mmHg according to the guidelines for prevention and treatment of hypertension in China (2010)^23^. Dyslipidemia of our participants was defined if their TC ≥ 6.22 mmol/L, HDL-C <1.04 mmol/L, LDL-C ≥ 4.14 mmol/L or TG ≥ 2.26 mmol/L according to the guidelines for the prevention and treatment of blood lipids in Chinese adults (2006)^24^.

### Specification of dietary patterns

Dietary patterns of adult residents in Jiangsu province were established by PCA method. To increase the comparability and interpretability of our findings, we adopted the same criteria as in the previous study (i.e., eigenvalueå 1, scree plot and the total variance of 43.94%) and retained four principal dietary factors (i.e., dietary patterns) into the current study^17,25,26^. We named each dietary pattern according to the food components included into the model obtained from PCA. Each dietary pattern was further divided into four subgroups by quartile of the factor scores (T1, T2, T3 and T4 group).

### Statistical analysis

Categorical variables and continuous variables were analyzed by *χ*^2^ test and Student’s T-test, respectively. The continuous variables were expressed as mean ± SD while the categorical variables were presented as frequency (the proportion or percentage). PCA method was used to build dietary patterns using the same approach of the previous study^17,25,26^, and multivariate logistic regression analysis was used to calculate the odds ratio (*OR*) and the 95% confidence interval (95% *CI*) for the association between dietary patterns and abnormalities blood glucose (IFG, IGT, IFG combined with IGT). All data were double entered into Epidata 3.5 and analyzed by SPSS 21.0.

### Ethics approval and consent to participate

This study protocol was approved by the ethical review committee of the Jiangsu Province Center for Disease Control and Prevention (the committee’s reference number: SL2015-B004-01). In this manuscript, we have not contained any individual person’s data in any form (including any individual details, images or videos). Written informed consent was obtained from all study participants.

## Results

### Basic characteristics of the study population

Overall, the prevalence of diabetes and prediabetes was 9.42% (791/8400) and 32.90% (2766/8400), respectively. The age-standardized prevalence of diabetes was 7.09%, which included both previously diagnosed diabetes and newly identified diabetes in the survey (7.56% among men and 6.71% among women). The age- standardized prevalence of prediabetes was 29.52% (32.45% among men and 27.11% among women).

A total of 7555 eligible adults were included in the dietary pattern analysis with 3408(*45.11%*) men and 4147 (*54.89%*) women. The mean age of all eligible participants was 51.27 ± 14.79 years old and age distribution was similar between men (51.47 ± 14.89 years) and women (51.71 ± 15.01 years). Gender, age, alcohol drinking, BMI, marital status, and so on were significantly correlated with the prevalence of prediabetes (*P* < *0.05*), the details were shown in Table 1.

**Table 1:** Characteristics of participants and the prediabetes.

		Number	Prediabetes[n(%)]	*χ*^2^	*P* value
Gender	Female	4147	1344 (32.4)	12.58	<0.001
	Male	3408	1234 (36.2)		
Age(years)	18–34	966	224 (23.2)	131.751	<0.001
	35–44	1586	457 (28.8)		
	45–54	1754	587 (33.5)		
	55–64	1778	689 (38.8)		
	≥65	1471	621 (42.2)		
Current smoker	No	5504	1860 (33.74)	0.979	0.322
	Yes	2051	718 (35.01)		
Alcohol drinking	No	4946	1644 (33.24)	4.98	0.026
	Yes	2609	934 (35.80)		
BMI^*^ (kg/m^2^)	≤18.49	194	43 (22.2)	136.687	<0.001
	18.50–23.99	3600	1030 (28.6)		
	24.00–27.99	2790	1062 (38.1)		
	≥28.00	965	442 (45.8)		
Marital status	Single	309	64 (20.7)	29.376	<0.001
	Married	7240	2514 (34.7)		
	Others	6	0 (0)		
Occupation	Farmers,forestry,herders and fishermen	2443	863 (35.4)	112.13	<0.001
	Technicians and machine operators	1287	334 (26.0)		
	Commerce	753	245 (32.5)		
	Enterpriser	130	56 (43.1)		
	Housework	1245	431 (34.6)		
	Retiree	700	338 (48.3)		
	Others	997	311 (31.2)		
Urban and rural	Urban	4307	1594 (37.0)	39.213	<0.001
	Rural	3248	984 (30.3)		
Hypertension	No	4600	1343 (29.2)	125.24	<0.001
	Yes	2955	1235 (41.8)		
Dyslipidemia^**^	No	5053	1590 (31.5)	48.939	<0.001
	Yes	2494	988 (39.6)		
Family history of diabetes	Yes	408	146 (35.8)	2.586	0.274
	No	6328	2135 (33.7)		
	Not clear	819	297 (36.3)		
Family history of Hypertension	Yes	2278	774 (34.0)	7.887	0.019
	No	4335	1445 (33.3)		
	Not clear	942	359 (38.1)		

Note: *6 participants have not the BMI information; **8 participants have not the blood lipid information.

### Determination of dietary patterns

**Figure 1:**
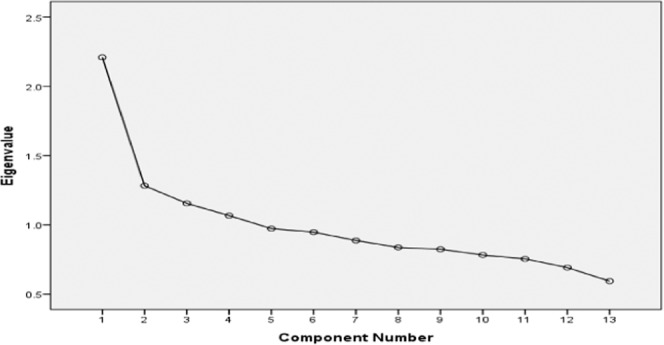
Scree plot for identification of dietary patterns by principal component analysis.

By PCA method we retained 4 principal components (factors) -dietary patterns explaining 43.939% of the diet variation (Fig. 1, Table 2). The Kaiser-Meyer-Olkin (KMO) index had a value of 0.710 (*P *< 0.001, Bartlett ball test), indicating the internal coherence of original variables was strong, and thus appropriate for the PCA. According to the preset criteria of variable selection (i.e., latent value >1 and the accumulation of total variance is 43.838%), four principal domains (i.e., dietary patterns) were selected from the PCA modeling. The food group factor loadings for each dietary pattern are shown in Table 2. Dietary pattern identified by Factor 1, labeled Meat pattern diet (the latent value is 2.209, the variance contribution is 16.990%, the cumulative variance contribution is 16.990%), was characterized by a high intake of poultry, beef and mutton, and pork. Dietary pattern identified by Factor 2, labeled Healthy diet (the latent value is 1.282, the variance contribution is 9.864%, the cumulative variance contribution is 26.854%), with food items of fresh fruits, vegetables, milk and eggs. Dietary pattern identified by Factor 3, labeled Traditional diet (the latent value is 1.155, the variance contribution is 8.882%, the cumulative variance contribution is 35.736%), mainly including rice and flour, vegetables, and beans. Dietary pattern identified by Factor 4, labeled Fried food with staple diet (the latent value is 1.066, the variance contribution is 8.204%, the cumulative variance contribution is 43.939%), mainly including the foods rich in fat and carbohydrate, such as fried food and tubers based fried food.

**Table 2:** The factors of dietary patterns.

Factor 1-Meat diet food factor		Factor 2-Healthy diet food factor		Factor 3-Traditional diet food factor		Factor 4-Fried food with staple diet food factor	
Poultry	0.651	Fresh fruit	0.517	Rice and flour	0.667	Fried food	0.556
Beef and mutton	0.548	Fresh vegetable	0.394	Fresh vegetable	0.529	Rice and flour	0.271
Pork	0.527	Milk products	0.401	Tuber	0.339	Tuber	0.227
Fish	0.485	Eggs	0.272	Bean products	0.198	Bean products	0.206
Egg	0.384	Tuber	0.065	Fish	0.067	Fresh fruit	0.050
Cake	0.259	Bean products	0.032	Fresh fruit	−0.086	Fresh vegetable	−0.181
Rice and flour	0.147	Fried food	−0.248	Milk products	−0.348	Pork	−0.154

### The relationship between dietary patterns and prediabetes

Participants with the Healthy diet pattern had the lowest prevalence of prediabetes (33.0%), followed by Traditional diet (34.1%) and Meat diet (35.4%), and the highest prevalence of prediabetes was found among those consuming Fried food with staple diet (36.9%) (Table 3). After adjustment for the confounders, the association between diet patterns and prediabetes retained significant for the Meat diet (*OR* = 1.229, 95% *CI:* 1.077–1.402, *P* < 0.01) and Fried food with staple diet (*OR* = 1.312, 95% *CI*: 1.110–1.551, *P* < 0.01).

**Table 3:** Multi-factor logistic regression analysis of personal dietary patterns and prediabetes.

Personal dietary patterns	Prediabetes N (%)	Crude model		Multivariate model^#^	
		*P* value	*OR*(95% *CI*)	*P* value	*OR*(95% *CI*)
Healthy diet	1081(33.0)	0.104		0.001	
Traditional diet	559(34.1)	0.384	1.057(0.933~1.199)	0.715	1.025(0.899~1.168)
Meat diet	638(35.4)	0.067	1.120(0.992~1.264)	0.002	1.229(1.077~1.402)
Fried food with staple diet	300(36.9)	0.035	1.188(1.013~1.395)	0.001	1.312(1.110~1.551)

Note: ^#^ adjusted for gender, age, hypertension, dyslipidemia, urban or rural, marriage, career, family history of Hypertension, family history of diabetes, alcohol drinker, and current smoker

The results of *χ*² test showed that there have been significantly different among the four subgroups quartiles (T1-T4 group) in prediabetes prevalence in Healthy diet, T1 group has the highest prevalence of prediabetes (Table 4), this phenomenon was not found in other diet patterns. Crude logistic regression analysis showed that the OR for the third quartile (T3 group) of Healthy diet was significantly associated with lowest prevalence of prediabetes (*OR* = 0.808, 95% *CI*: 0.706–0.924, *P* < 0.01) compared with the other groups. A similar significant association was retained in multivariate logistic regression model after adjusting for other major confounder factors including gender, age, hypertension, dyslipidemia, current smoker, alcohol drinker, BMI, urban or rural, marriage career, family history of diabetes, family history of hypertension (*OR* = 0.745, 95% *CI*: 0.645–0.860, *P* < 0.01), as shown in Table 5.

**Table 4:** Comparison of the prevalence of prediabetes among different ranges of dietary patterns.

Dietary pattern	Meat diet	Healthy diet	Traditional diet	Fried food with staple diet
T1	630(33.4)	696(37.0)	667(35.4)	655(34.7)
T2	628(33.3)	636(33.7)	656(34.9)	614(32.6)
T3	649(34.5)	607(32.2)	626(33.2)	629(33.4)
T4	671(35.6)	639(34.0)	629(33.4)	680(36.1)
*χ*²	2.977	10.376	3.07	5.701
*P*	0.395	0.016	0.381	0.127

**Table 5:** Multi-factor logistic regression analysis of the Healthy dietary patterns and prediabetes.

dietary pattern		Prediabetes ^N (%)^	Crude model		Multivariate model^#^	
			*P* value	OR(95% CI)	*P* value	OR(95% CI)
Healthy diet	T1	696(37.0)	0.016		0.001	
	T2	636(33.7)	0.032	0.864(0.756–0.988)	0.015	0.842(0.733–0.967)
	T3	607(32.2)	0.002	0.808(0.706–0.924)	<0.001	0.745(0.645–0.860)
	T4	639(34.0)	0.042	0.870(0.761–0.995)	0.005	0.813(0.703–0.940)

Note: #adjusted for gender, age, Hypertension, dyslipidemia, alcohol drinker, BMI, urban or rural, marriage, career, family history of Hypertension, current smoker, family history of diabetes.

The prevalence of isolated IFG was the highest (28.5%) among the prediabetes, and the prevalence of isolated IGT was the lowest. Table 6 shows the *O*R and 95% *CI* for the associations between dietary patterns and indexes of prediabetes. Results from multivariate logistic regression analysis showed a positive association between Meat diet and isolated IFG (*OR* = 1.227, 95% *CI*:1.070–1.406, *P* < 0.01); Fried food with staple diet was positively associated with IFG combined with IGT (*OR* = 1.735, 95% *CI*: 1.184–2.543, *P* < 0.01), whereas none of the dietary patterns was found to be associated with the presence of IGT (Table 6).

**Table 6:** Multi-factor logistic regression analysis of personal patterns and isolated IFG/IGT, IFG combined IGT.

Dependent variable	Prediabetes N (%)	Personal dietary patterns	Crude model		Multivatiate model^#^	
			*P* value	*OR*(95% *CI*)	*P* value	*OR*(95% *CI*)
Isolated IFG	2156(28.5)	Healthy diet	0.033		0.029	
		Traditional diet	0.662	1.031(0.900–1.181)	0.630	1.034(0.902–1.185)
		Meat diet	0.004	1.222(1.066–1.400)	0.003	1.227(1.070–1.406)
		Fried food with staple diet	0.372	1.084(0.908–1.296)	0.361	1.086(0.909–1.298)
Isolated IGT	174(2.3)	Healthy diet	0.225		0.225	
		Traditional diet	0.795	0.945(0.616–1.449)	0.760	0.935(0.609–1.436)
		Meat diet	0.804	1.056(0.686–1.627)	0.813	1.054(0.683–1.625)
		Fried food with staple diet	0.064	1.533(0.976–2.408)	0.067	1.527(0.971–2.401)
IFG combined IGT	248(3.3)	Healthy diet	0.012		0.012	
		Traditional diet	0.774	0.951(0.676–1.339)	0.774	0.951(0.675–1.339)
		Meat diet	0.659	0.921(0.638–1.329)	0.654	0.919(0.637–1.328)
		Fried food with staple diet	0.005	1.739(1.187–2.547)	0.005	1.735(1.184–2.543)

Note: ^#^Adjusted for gender, age, Hypertension, dyslipidemia, alchol drinker, BMI, urban or rural, marriage, career, family history of Hypertension, current smoker, family history of diabetes.

## Discussion

This population-based survey revealed that 32.4% of male and 36.2% of female participants had IFG and/or IGT in Jiangsu province in China, with the prevalence of prediabetes of 34.2% among Chinese adults in Jiangsu Province. In 2010 China Noncommunicable Disease Surveillance, it was estimated that the prevalence of prediabetes was 50.1% (95% *CI*: 49.7%- 50.6%) in Chinese adults (18 years old or over), 52.1% (95% *CI*: 51.5%-52.7%) in men and 48.1% (95% *CI*: 47.6%-48.7%) in women^27^. The national prevalence of prediabetes in Chinese adults was significantly higher than that in Jiangsu Province, but the national prediabetes level in China includes glycated hemoglobin (HbA1c) diagnosis. Therefore, the prevalence of prediabetes in Jiangsu Province and China was severe and increased rapidly compared with the previously reports^2,28^.

The investigation method of dietary intake in this research including food frequency table method to collect the dietary data within the past year, which can reflect the daily dietary intake status of the participants and is a better measurement than those obtained using three days weighting method^29^. In this study, we used the method of PCA to determine the dietary patterns in a provincially representative sample and obtain four dietary patterns in Jiangsu Province adults in China, which were Meat diet, Healthy diet, Traditional diet and Fried food with staple diet. The Kaiser-Meyer-Olkin (KMO) index had a value of 0.710 (*P* <0.001, Bartlett ball test) in this study, indicating the internal coherence of original variables was strong, and thus appropriate for the PCA^17^. This dietary pattern’s result in our study was basically the same with the Fujian provincially representative results^29^, but had something different from other studies, such as Zhu Ling from Luoyang city, Henan Province, China;^30^ Zhu Ting from Guangxi Zhuang Autonomous Region, China^25^. The diversity of dietary patterns in China can be seen in some studies, and there are certain differences in different regions in China^29^. In our study, the potential confounders for dietary patterns were included in the logistic regression model, so the results of our analysis have a better comparability for all of the previous knowledge and the other similar studies.

We found that the Healthy diet in this study included fresh fruits, vegetables, milk and eggs, which is very similar to the Dietary Approaches to Stop Hypertension (DASH) diet that is evident to effectively reduce blood pressure^31^ and control of BMI and insulin metabolism^32^. The Healthy diet in this study is a potential protective factor for people with prediabetes, as the prevalence of prediabetes in this group is the lowest compared with other dietary patterns. Our finding was consistent with those of other countries in which a beneficial effect of higher intake of fresh fruit was indicated for the primary and secondary prevention of diabetes^9–14^. A dietary pattern characterized by fruits, vegetables, and soy has also been associated with a lower risk of diabetes and lower prevalence of IFG in nonsmoking Chinese^33,34^. However, most epidemiology studies were focused on the diabetes, only a few limited epidemiological studies had examined the relationship between dietary patterns and prediabetes^35–38^. Meat diet was reported in Meilin Zhang’s study and showed a positive association with the increased prevalence of isolated IFG which was consistent with our findings;^14^ however, our study is the first to systemically investigate the dietary patterns using PCA and then explored the associations with more indexes of prediabetes, which are regarded as the major merit superior to the previous studies.

In our study, Meat diet was positively associated with isolated IFG while the Fried food with staple diet was positively related to IFG combined with IGT. These findings suggest that the effects of dietary patterns on isolated IFG are greater than that of the isolated IGT, which indicates that high fat food may worsen the blood glucose control. The animal offal is generally high in saturated fatty acid, cholesterol, iron, and selenium, and desserts that contribute importantly to the glycemic load, and which in turn is associated with an increased risk of IFG;^14^ nevertheless, the potential biological mechanisms still need to be further studied.

However, this study also has some limitations. First, PCA is based on the collected dietary information, and used only the covariance matrix of predictors to get the dietary pattern without taking the outcome variable into consideration, so the dietary patterns may not be the most optimal^26,39^. Second, according to the ADA 2010 criteria, prediabetes was defined as the HbA1c concentrations between 5.7% and 6.4% in participants without a prior diabetes diagnosis. However, in our study, we did not examine the HbA1c level, that will underestimate the prevalence of prediabetes, therefore, we were not able to determine the association between dietary patterns and the prevalence of prediabetes used the HbA1c criteria. Besides, due to the cross-sectional nature of our study and potential reverse causation bias, associations between some risk factors and prediabetes were in unexpected directions.

In conclusion, this study demonstrated that Meat diet with high fat food (i.e poultry, beef, pork) may increase the risk of prediabetes (especially the isolated IFG), while Healthy diet with fresh fruit, fresh vegetable, tuber, fish and so on may potentially benefit to prediabetes. Our study advocates a Healthy dietary pattern including a low Meat and high Fresh food (fruit, vegetable, tuber, fish) should be promoted in both male and female general population with the ultimate goal to reduce the prevalence of prediabetes and safeguard the population health.
